# Effect of Hemodynamic Monitoring Systems on Short-Term Outcomes after Living Donor Liver Transplantation

**DOI:** 10.3390/medicina60071142

**Published:** 2024-07-16

**Authors:** Hakan Kilercik, Sami Akbulut, Sema Aktas, Utku Alkara, Sinasi Sevmis

**Affiliations:** 1 Department of Anesthesiology and Reanimation, Gaziosmanpasa Hospital, Istanbul Yeni Yuzyil University Faculty of Medicine, 34010 Istanbul, Turkey; hakankilercik@hotmail.com; 2 Department of Surgery and Liver Transplant Institute, Inonu University Faculty of Medicine, 44280 Istanbul, Turkey; 3 Department of Surgery and Organ Transplantation, Gaziosmanpasa Hospital, Istanbul Yeni Yuzyil University Faculty of Medicine, 34010 Istanbul, Turkey; semaakt@gmail.com (S.A.); ssevmis@yahoo.com (S.S.); 4 Department of Radiology, Gaziosmanpasa Hospital, Istanbul Yeni Yuzyil University Faculty of Medicine, 34010 Istanbul, Turkey; utkualkara@yahoo.com

**Keywords:** living donor liver transplantation, intraoperative management, hemodynamic monitoring, pressure recording analytical method, pulse index continuous cardiac output

## Abstract

*Background and Objectives*: To evaluate the effects of the pulse index continuous cardiac output and MostCare Pressure Recording Analytical Method hemodynamic monitoring systems on short-term graft and patient outcomes during living donor liver transplantation in adult patients. *Materials and Methods*: Overall, 163 adult patients who underwent living donor liver transplantation between January 2018 and March 2022 and met the study inclusion criteria were divided into two groups based on the hemodynamic monitoring systems used during surgery: the MostCare Pressure Recording Analytical Method group (*n* = 73) and the pulse index continuous cardiac output group (*n* = 90). The groups were compared with respect to preoperative clinicodemographic features (age, sex, body mass index, graft-to-recipient weight ratio, and Model for End-stage Liver Disease score), intraoperative clinical characteristics, and postoperative biochemical parameters (aspartate aminotransferase, alanine aminotransferase, total bilirubin, direct bilirubin, prothrombin time, international normalized ratio, and platelet count). *Results*: There were no significant between-group differences with respect to recipient age, sex, body mass index, graft-to-recipient weight ratio, Child, Model for End-stage Liver Disease score, ejection fraction, systolic pulmonary artery pressure, surgery time, anhepatic phase, cold ischemia time, warm ischemia time, erythrocyte suspension use, human albumin use, crystalloid use, urine output, hospital stay, and intensive care unit stay. However, there was a significant difference in fresh frozen plasma use (*p* < 0.001) and platelet use (*p* = 0.037). *Conclusions*: The clinical and biochemical outcomes are not significantly different between pulse index continuous cardiac output and MostCare Pressure Recording Analytical Method as hemodynamic monitoring systems in living donor liver transplantation. However, the MostCare Pressure Recording Analytical Method is more economical and minimally invasive.

## 1. Introduction

Liver transplantation (LT) is one of the most important treatment modalities for liver diseases, including chronic liver disease, acute liver disease, primary liver cancer, and some inherent pediatric diseases. LT is associated with hyperdynamic circulation, including a high resting cardiac output (CO) and low systemic vascular resistance. Hyperdynamic conditions can coexist with a relative hypovolemic status during LT surgery because of intraoperative reperfusion associated with peripheral autonomic neuropathy in patients with cirrhosis. During LT, some problems can induce significant circulatory instability and liver damage, such as bleeding, circulatory volume fluctuations, vascular clamping/unclamping, and large fluid shifts [1,2,3]. Considering the possibility of significant cardiovascular changes during LT surgery in association with the disease process, vigilant and interactive hemodynamic monitoring is crucial to assess preload, afterload, and cardiac function [4]. Early diagnosis and goal-directed therapy using hemodynamic monitoring are the best methods for coping with these issues. In most institutions, classic hemodynamic measurements such as invasive arterial pressure, heart rate and rhythm, and CO measured using a pulmonary artery catheter (PAC) are still used [3,4,5]. The main principle of CO monitoring is an invasive thermodilution technique using PAC. However, the use of PAC has been debated because of its invasiveness and the possibility of serious complications. As such, some minimally invasive techniques, such as pulse index continuous cardiac output (PiCCO) plus PAC and the MostCare Pressure Recording Analytical Method (MostCare PRAM) plus PAC, have been introduced for monitoring [6].

However, there are limited data on the effects of these minimally invasive cardiopulmonary monitoring techniques on early graft function and hemodynamic problems after LT. The incidence of early allograft dysfunction after LT is close to 25%, and there are several risk factors for early allograft dysfunction; these include donor age, race, cause of death, height, weight, donor type, total ischemic time (from donor cross-clamp time to recipient reperfusion time), recipient age, race, diagnosis, Model for End-stage Liver Disease (MELD) score, and United Network for Organ Sharing (UNOS) status [7].

This study aimed to evaluate the effects of the hemodynamic monitoring systems PiCCO and MostCare on short-term graft and patient outcomes of adult patients undergoing living donor liver transplantation (LDLT).

## 2. Materials and Methods

### 2.1. Study Design and Population

This retrospective study evaluated 174 adult LT candidates who underwent LDLT at the Department of Organ Transplantation, Gazisomanpasa Hospital, Yeni Yuzyil University Faculty of Medicine, Istanbul, Turkey, between January 2018 and March 2022. Among them, 11 recipients were excluded because of primary graft dysfunction (*n* = 2), sepsis (*n* = 4), hepatic artery thrombosis (*n* = 4), or pulmonary vein thrombosis (*n* = 1) within the first week. Finally, 163 adult patients were included. All recipients underwent surgery under general anesthesia and invasive intraoperative monitoring by the same team experienced in solid organ transplantation anesthesiology. All surgeries were performed by the same team of surgeons using the modified piggyback technique, and neither venovenous bypass nor a temporary portocaval shunt was employed [8].

This study analyzed cohort data (demographic and clinicopathological characteristics and peri- and postoperative data, including information on operative and postoperative clinical courses) recorded in electronic databases. Biochemical measures including platelet (PLT) count, total bilirubin, and direct bilirubin were aspartate aminotransferase (AST) level, alanine aminotransferase (ALT) level, prothrombin time (PT), international normalized ratio (INR), obtained from each patient at the day of surgery (postoperative day (POD) 0) and PODs 1, 3, and 7. LT recipients were divided into two groups according to the intraoperative hemodynamic monitoring system used: the PiCCO group (*n* = 90) and the MostCare PRAM group (*n* = 73).

### 2.2. Anesthesia Management and Intraoperative Hemodynamic Monitoring

Our institutional anesthesia protocol was applied to all LDLT recipients as follows: anesthesia was induced with propofol, fentanyl, midazolam, and rocuronium and was maintained using 1–2 vol% sevoflurane, 40–50% oxygen/air, and continuous infusion of rocuronium and remifentanyl. All recipients were intubated and mechanically ventilated using endotracheal tubes (Drager Primus; Drager Medical Inc., Telford, TX, USA). The ventilator settings were initially set using a constant tidal volume of 8–10 mL/kg and a respiratory rate of 10–12 breaths/min based on the patient’s biometric information. This was then adjusted based on measurements of respiratory and blood gas parameters to maintain a constant end-tidal carbon dioxide tension of 30–35 mmHg. During the surgical procedure, a multiparameter bedside monitor (Draeger Infinity Kappa, Lübeck, Germany) was used to monitor heart activity (five-lead electrocardiography), oxygen saturation (pulse oximetry), partial pressure of carbon dioxide (capnography), invasive blood pressure, internal temperature, and urine output.

Isotonic and balanced crystalloid solutions were used to replace the intravascular volume (10 mL/kg/h) according to electrolyte demands. The amount of human albumin solution (5%) administered as a volume expander was determined by the degree of hypoalbuminemia (1–1.5 g/kg). Packed red blood cells (PRBCs) were transfused to maintain the hemoglobin level at 8 g/dL. Following laboratory data collection and surgical field examination, fresh frozen plasma (FFP) and PLT transfusions were performed. Furosemide (0.2–0.5 mg/kg) or mannitol 20% (0.3 g/kg) was given, as needed, to achieve a mean urine output of 1 mL/kg/h. Continuous arterial blood pressure, central venous pressure (CVP), and pulmonary arterial pressure (PAP) were recorded using invasive monitoring. All percutaneous intravascular interventions were performed under ultrasonography supervision.

Owing to the need for frequent blood sampling and continuous invasive blood pressure monitoring during the lengthy procedure, the radial and femoral arteries of all patients were cannulated jointly. The radial artery cannula (3.5-Fr 8 cm arterial Leadercath Vygon, Ecouen, France) was placed under sedation before induction of anesthesia, and the arterial pressure was determined using a standard anesthesia monitor. Therefore, blood samples for blood gas, electrolyte, and coagulation analyses were drawn from the radial artery at planned time points and according to the patient’s clinical profile. After the induction of anesthesia, the femoral artery cannula was placed and used for thermodilution and pulse counter analyses in both groups. Because central aortic pressure monitoring is regarded as more precise, especially during hemodynamic instability, this cannula was selected for radial artery monitoring. A 4-way 8.0-Fr CVP catheter was inserted through the right or left internal jugular vein, and a 7-Fr PAC (Swan-Ganz Edwards Lifesciences, Irvine, CA, USA) was introduced through the right internal jugular vein in combination with an 8.5-Fr large-bore introducer in all patients [2,7]. Without CO data, we used PAC to monitor only the right-heart filling pressure, PAP, and pulmonary arterial wedge pressure (PAWP; also known as pulmonary wedge pressure).

Intravenous fluids, blood products, medicines, and interventions were administered according to clinical needs to maintain hemodynamic, metabolic, and coagulation homeostasis. Catecholamines were utilized to stabilize circulation during graft reperfusion, if necessary. Following the induction of anesthesia, antimicrobial prophylaxis was administered, and additional doses were administered according to the duration of the LT procedure. During the anhepatic phase, all the recipients received 10 mg/kg methylprednisolone. A warming blanket, heat and moisture exchanger, room temperature thermostatically controlled at 24 °C, vinyl arm wraps, and a fast fluid warmer were used to maintain the patients’ body temperatures above 35 °C. The attending anesthesiologist decided the hemodynamic parameters, volume resuscitation, transfusion management, and cardiovascular-related medication therapy for each patient after consulting the surgical team. The graft was implanted using the piggyback surgical technique that preserved caval flow by partially clamping the inferior vena cava during the hepatic vein–inferior vena cava anastomosis. If this method was not possible, the inferior vena cava was entirely constricted. Venovenous bypass is rarely used in our practice, and temporary portocaval shunts are never used. The graft was reperfused after the portal vein anastomosis by unclamping the hepatic and portal veins. Subsequently, hepatic artery and biliary anastomoses were performed.

### 2.3. Hemodynamic Monitoring for the PiCCO Group

The patients were monitored for CO using a PiCCO2 device (Pulsion Medical Systems, Munich, Germany). Calibrated through transpulmonary thermodilution (TPTD), this device provides beat-to-beat real-time analysis of the femoral arterial pressure curve [2]. The TPTD performed with this system requires central venous cannula (with Pulsion PiCCO injectate temperature sensor housing device used) to inject cold saline and a specific 5-Fr 20 cm thermistor-tipped femoral arterial cannula (PiCCO Catheter, PV2015L20-A^®^; Pulsion Medical System).

The TPTD measurement for pulsed PiCCO2 was performed in sets of three bolus injections of 15 mL cold isotonic saline at a temperature lower than 10 °C through the central venous catheter, irrespective of the ventilator cycle. The results were accepted to show a consistent shape of the thermodilution curve and had less than 15% variation from previous measurements. Intermittent bolus TPTD measurements for calibration were performed specifically at three time points: after induction, after portal vein clamping during the anhepatic phase, and 15 min after reperfusion. To avoid variations, the injection was always performed by the same clinician when the patient was in a stable hemodynamic condition (mean arterial pressure (MAP), 70 mmHg; sinus rhythm) with a constant rate of intravenous fluid infusion and drugs. Surgical procedures were paused between TPTD calibrations. After the TPTD calibration, starting continuous estimation of CO, beat-by-beat continuous CO estimation was provided using the arterial pulse wave.

Recipients in the PiCCO group were managed based on parameters obtained with the Pulsion PiCCO device monitoring system. These included the MAP (>65 mmHg), cardiac index (CI, 3–5 L/min/m^2^), stroke volume index (SVI, 40–60 mL/m^2^), systemic vascular resistance index (SVRI, 1700–2400 dyn*s*cm-5*m^2^), global end-diastolic volume index (global end-diastolic volume (GEDV), 680–800 mL/m^2^), extravascular lung water index (EVLWI, 3–7 mL/kg), stroke volume variation (SVV, <10%), global ejection fraction (GEF, 25–35%), and cardiac function index (CFI, 4.5–6.5 min^−1^) [9,10,11].

### 2.4. Hemodynamic Monitoring for the MostCare PRAM Group

Patients were monitored using the PRAM (PRAM-CO) MostCare Up (MostCare; Vytech Health, Padova, Italy) device for CO with arterial pulse wave counter analysis using the pressure-recording analytical method. A femoral arterial cannula (3.5-Fr 8 cm arterial Leadercath, Vygon, Ecouen, France) was connected to the device transducer. It was used to analyze arterial pressure waveform data over 30 s intervals, using a recalibration interval of 1 min. After setting the arterial pressure transducer system to zero and before each measurement, the arterial waveform signal fidelity was checked visually using a fast flush test to assess the adequacy of the damping of the arterial shape [11].

Recipients in the MostCare PRAM group were managed according to the parameters obtained with the PRAM (PRAM-CO) monitoring system, including MAP (>65 mmHg), CI (2.6–3.8 L/min/m^2^), SVI (35–45 mL/m^2^), SVRI (1600–2400 dyn*s*cm-5*m^2^, SVV (<10%), maximal slope of the systolic upstroke (dP/dtmax 0.9–1.3 mmHg/ms), and oxygen delivery index (DO2I 500–600 mL/min/m^2^) (normal values were obtained from the manufacturer’s calibration) [11,12,13].

The PAC was inserted via the introducer sheath into the right internal jugular vein and advanced to a wedged position under the guidance of a pressure curve. In addition to the PiCCO and MostCare values, PAP and PAWP values monitored instantly on the PA catheter were considered in the treatment of both groups. Hemodynamic indices and parameters were evaluated according to general standards to guide goal-directed fluid and medication therapies. Fluid replacement and cardiovascular medications were administered to patients who were managed such that the monitoring parameters remained within the specified limits based on their clinical condition.

### 2.5. Study Protocol and Ethics Committee Approval

This study involving human participants was conducted according to the ethical standards of the institutional and national research committee and the 1964 Helsinki Declaration and its later amendments or comparable ethical standards. Ethical approval was obtained from the Inonu University Institutional Review Board (IRB) for noninterventional studies (Approval No: 2023/5354). The Strengthening the Reporting of Observational studies in Epidemiology (STROBE) guideline was utilized to assess the likelihood of bias and overall quality for this study [14].

### 2.6. Statistical Analysis

The Shapiro–Wilk test was used to assess normality among the studied variables. Continuous variables are presented as medians (95% confidence intervals), while categorical variables are reported as numbers (n) and percentages (%). Between-group comparisons were performed using the Mann–Whitney-U test. Pearson’s chi-squared test was used to compare categorical variables. The nonparametric Friedman test was used for repeated measurements within groups. The Statistical Package for the Social Sciences (IBM SPSS Statistics for Windows, version 25.0, Armonk, NY, USA) was used for all statistical analyses. Statistical significance was set at *p* < 0.05.

## 3. Results

The cohort involved 110 (67.5%) males and 53 (32.5%) females aged 18–73 years. The sociodemographic, clinical, surgical, and intraoperative monitoring characteristics of the PiCCO and MostCare PRAM groups are shown in Table 1. There were no significant between-group differences in body mass index (*p* = 0.806), donor age (*p* = 0.748), Child (*p* = 0.074), MELD score (*p* = 0.175), ejection fraction (*p* = 0.123), systolic PAP (*p* = 0.068), surgery time (*p* = 0.058), anhepatic phase (*p* = 0.460), cold ischemia time (*p* = 0.518), warm ischemia time (*p* = 0695), erythrocyte suspension use (*p* = 0.548), human albumin use (*p* = 0.103), crystalloid use (*p* = 0.479), urine output (*p* = 0.513), GRWR (*p* = 0.275), hospital stay (*p* = 0.897), and ICU stay (*p* = 0.060). However, FFP use (*p* < 0.001) and PLT use (*p* = 0.037) were significantly different between the two groups. Changes in biochemical test results on POD 0 and on POD 1, POD 3, and POD 7 after LT are summarized in Table 2. There were no significant differences between the PiCCO and MostCare PRAM groups with respect to AST level, ALT level, total bilirubin, and direct bilirubin levels measured. Although there were statistically significant differences between the PiCCO and MostCare PRAM groups in terms of INR and PT values that were measured in POD 0 and POD 1, this difference was not seen in POD 3 and POD 7.

There was no significant difference in AST levels between the two groups (*p* = 0.680). The change in AST level from POD 0 to POD 1 was not significantly different between the groups (*p* = 0.226), but that from POD 0 to POD 3 was significantly different, with AST levels decreasing by 55% and 66% in the PiCCO and MostCare PRAM groups, respectively (*p* = 0.014). In addition, the change in AST level from POD 0 to POD 7 was significantly different between the groups, with the levels decreasing by 77% in the PiCCO group and by 82% in the MostCare PRAM group (*p* = 0.046). For PT, the mean value of POD 0 was significantly higher in the MostCare PRAM group, at 21.6, than in the in the PiCCO group, at 20.0 (*p* = 0.003). The change in PT value from POD 0 to POD 3 was different between the two groups, with the decrease being significantly higher in the MostCare PRAM group than in the PiCCO (21.5% vs. 14.8%, *p* = 0.012). For the change from POD 0 to POD 7, the PT value decreased by 32% in the PiCCO group and by 36.8% in the MostCare PRAM group (*p* = 0.010).

**Figure 1:**
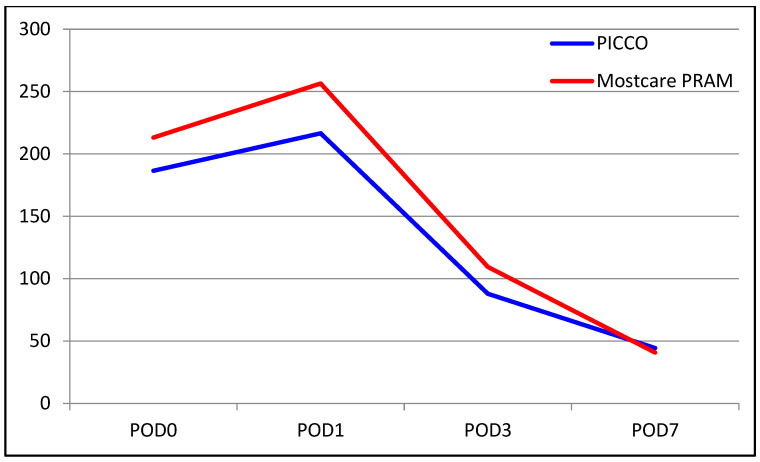
Graphical view of the change in AST level in groups over time.

**Figure 2:**
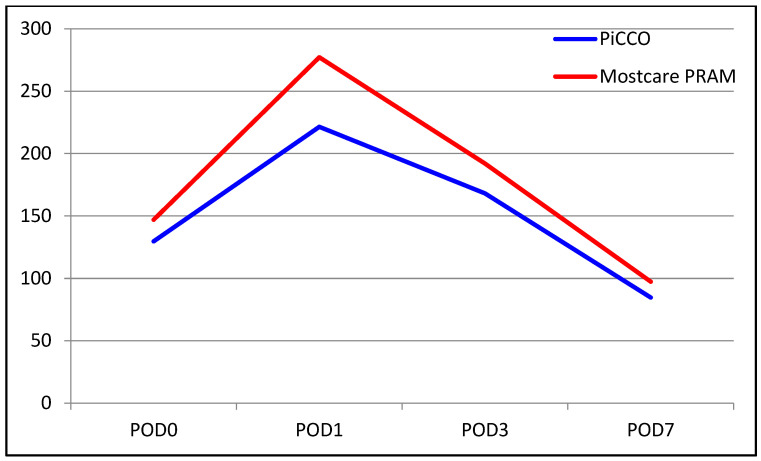
Graphical view of the change in ALT level in groups over time.

**Figure 3:**
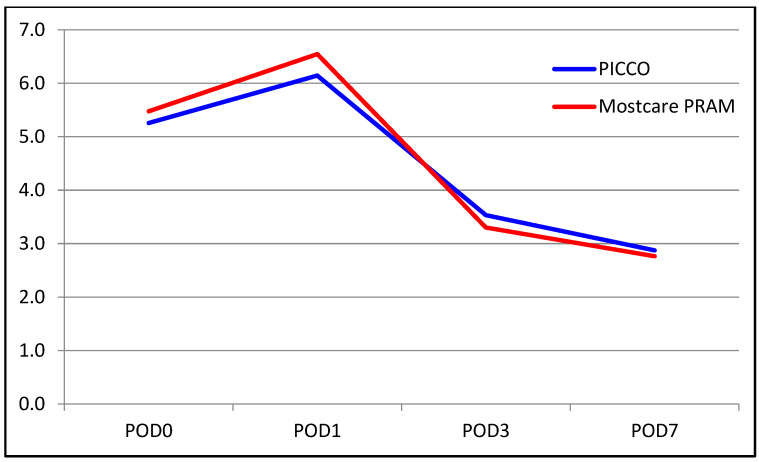
Graphical view of the change in total bilirubin level in groups over time.

**Figure 4:**
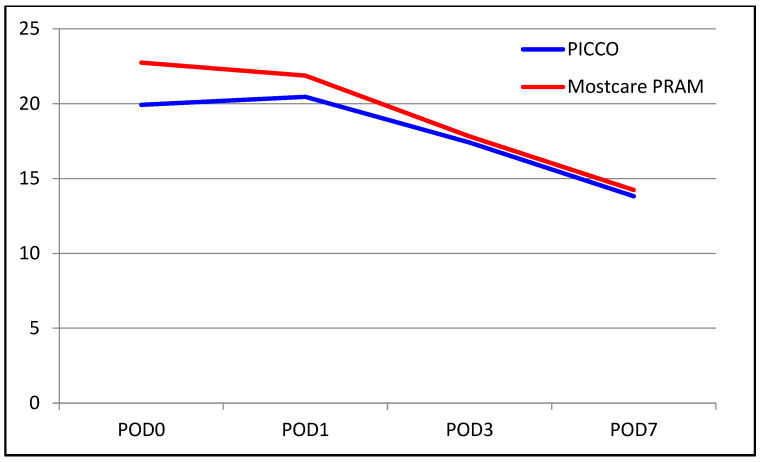
Graphical view of the change in prothrombin time in groups over time.

**Figure 5:**
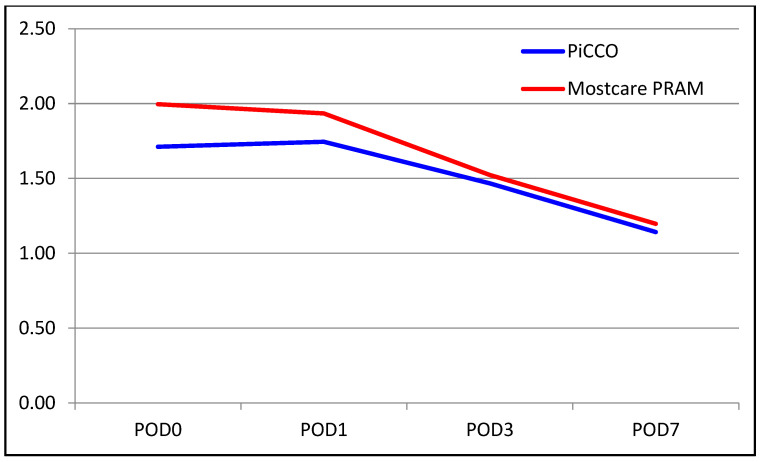
Graphical view of the change in INR level in groups over time.

**Figure 6:**
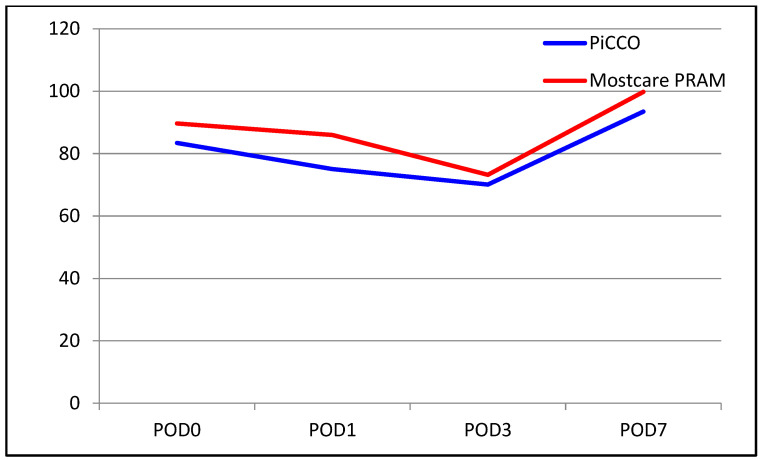
Graphical view of the change in platelet count in groups over time.

The median INR level was 1.70 in the PiCCO group and 1.8 in the MostCare PRAM group. The median INR level on POD 0 was higher in the MostCare PRAM group (*p* = 0.005). There were no significant between-group differences in the change from POD 0 to POD 1 (*p* = 0.350). However, significant differences were found for the change from POD 0 to POD 3, with the decrease in INR level being significantly higher in the MostCare PRAM group than in the PiCCO group (22% vs. 17%, *p* = 0.015). Similarly, the change from POD 0 to POD 7 was significantly different between the groups, with a decrease of 33% in the PiCCO group and 39% in the MostCare PRAM group (*p* = 0.033). A comparison of liver function test results between the groups are shown in Table 2 and Figure 1, Figure 2, Figure 3, Figure 4, Figure 5 and Figure 6.

## 4. Discussion

Long-term survival has been achieved in LT thanks to advances in surgical techniques, monitoring in the perioperative period, effective intensive care unit management, use of effective immunosuppressive agents with low side effects, management of complications with minimally invasive interventions, and close follow-up [15,16]. One of the most important steps for minimizing perioperative complications, early recognition of potential life-threatening complications, and achieving successful postoperative results is optimal intraoperative hemodynamic monitoring [15,16]. Minimally invasive cardiopulmonary monitoring techniques have been introduced, but there are limited data on their effects on early graft function and hemodynamic outcomes after LT. The current study found no significant differences between PiCCO and MostCare PRAM with respect to perioperative hemodynamic parameters, complications, and postoperative clinical and biochemical parameters. This finding indicates that neither system is superior to the other with respect to patient outcomes. Continuous monitoring of CI, CO, and MAP was achieved using both the PiCCO and MostCare PRAM systems. Hemodynamic management was achieved by monitoring the transdilution parameters GEDV index and intrathoracic blood volume index (ITBVI) as preload indicators, GEF and CFI as cardiac contractility indicators, SVV as a fluid responsiveness and dynamic variable, SVRI as an afterload parameter, and EVLW as a lung parenchymal fluid predictor with PiCCO, in addition to CO and PAP data [9,16,17]. Hemodynamic management with the MostCare PRAM device included SVRI, SVV, pulse pressure variation (PPV) values, maximal slope of systolic upstroke (dP/dtmax) as a contractility indicator, and cardiac cycle efficiency parameters as cardiac function efficiency parameters, in addition to MAP, CI, and PAP [18,19].

LT is generally performed in patients with chronic liver disease, and most patients undergo hemodynamic changes during the transplantation period. These cirrhosis-related changes are known as the hyperdynamic state, which is characterized by increased blood volume, high CO levels, and low total peripheral resistance [20]. Inadequate or variable blood pressure and low-flow conditions are common perioperative hemodynamic abnormalities. Graft dysfunction, prolonged dependence on mechanical ventilation, severe renal impairment, sepsis, and an increase in post-transplant morbidity and mortality are all attributed to these occurrences [21]. Therefore, hemodynamic alterations are among the most important factors affecting early graft function [21].

The main purpose of both PiCCO and MostCare PRAM as hemodynamic monitoring systems are to maintain optimal cardiovascular and pulmonary functions during LT, provide adequate portal and arterial blood flow to the liver, and maintain the pressure in the inferior vena cava within acceptable limits for liver drainage. Graft dysfunction or delayed graft function is associated with poor graft perfusion caused by substantial systemic or portal venous hypotension. Even a minor systemic circulatory disorder can exert a significant impact on splanchnic circulation and local graft circulation. Excessive vasoconstriction is harmful in the post-transplant setting, where maintaining microvascular flow and avoiding graft destruction are the key concerns. Hepatocyte hypoxia can be caused by passive venous congestion owing to low CO levels or a reduction in hepatic arterial and/or portal venous flow. An increase in liver transaminases and bilirubin and a decrease in albumin and coagulation factors are classical laboratory findings suggesting chronic generalized liver dysfunction. Critical laboratory and clinical parameters can be improved within days of restoring appropriate systemic hemodynamics and ameliorating graft microcirculatory impairment [22,23].

Although PAC remains the gold standard for monitoring LT recipients, its use is decreasing owing to its invasive nature and potential technical complications [2]. CO characteristics can be assessed intermittently or continuously using PAC. The accuracy of the CO parameters derived from PAC is determined by various factors, including the injectate temperature, volume, and speed in relation to the respiratory cycle. Large volumes of fluids that may be provided to compensate for the significantly hypotensive state and the cold preservation fluid mixed with blood generate temperature fluctuations during the reperfusion period of LT, which might contribute to an additional underestimation of the real CO. Despite the high correlation observed following LT, this approach has disadvantages similar to those of intermittent thermodilution, including poor accuracy during reperfusion and cross-clamping. Traditional estimates of intravascular volume status and cardiac filling pressures have poor correlations with changes in CO, and the more recently employed dynamic fluid responsiveness measures have shown significant shortcomings [24].

The capacity to monitor pulmonary pressure is one of the reasons why PAC catheters are still used in the follow-up of liver transplantation patients [2]. Meanwhile, patients with mild or moderate pulmonary hypertension are at an increased risk of perioperative morbidity and mortality. The preservation of graft function requires prompt management of acute increases in PAP during the intraoperative period in patients with preoperatively treated portopulmonary and borderline pulmonary hypertension. A lower CVP during the anhepatic and reperfusion phases has been associated with a lower requirement for blood products and better surgical circumstances [4].

A thermodilution catheter was inserted into the femoral artery using the PiCCO system. CO was determined by constructing a thermodilution curve from blood temperature variations caused by a bolus of cold saline delivered to the central venous access. Following this initial calibration, the device calculates CO on a continuous basis using arterial pulse contour analysis and offers information on the patient’s preload, afterload, myocardial contractility, CO, and EVLWI. In this study, CO, EVLW, GEDV, ITBVI, CFI, and GEF were calculated using the transcardiopulmonary thermodilution indicator–time curve. Continuous estimation of stroke volume, CO, PPV, SVV, and a left ventricular contractility index was performed via pulse contour analysis [9].

The PiCCO system algorithm for calculating the CO from an arterial waveform is based on a calibration factor that reflects the compliance and resistance of the arterial tree at the time of calibration. PiCCO levels may be affected by larger changes in arterial tone and pressure. In the event of consistent increases in the SVRI or acute blood loss, recalibrating the PiCCO is highly recommended. A study by Yamashita and colleagues [25] showed that when systemic vascular resistance was reduced dose dependently by prostaglandin E1 infusion, PiCCO underestimated CO by up to 40% when compared to the intermittent thermodilution CO method. One of the important advantages of the MostCare PRAM system is that it does not require external calibration. A recent systematic review comparing five popular systems for arterial pulse contour showed that CO was underestimated in all systems except PRAM, while it was overestimated in the MostCare PRAM system [26]. In MostCare PRAM, which is considered the least invasive device, CO is derived from the arterial waveform. It provides CO measurements based on arterial pulse contour analysis in conjunction with demographic patient data without the requirement for an independent calibration procedure or thermal dilution. CO can be measured using a sensor with a standard arterial line. This system is popular owing to its ease of setup and the fact that it only requires an arterial line to acquire CO, despite its significant limitations.

As the technology and precision of minimally invasive monitoring systems advance, well-designed prospective trials are required to evaluate their value and impact on postoperative clinical outcomes. The best strategy to improve performance is probably not to choose the “one and only” option. The integration of data from different monitoring systems relevant to clinical situations remains the only approach to properly manage hemodynamic instability. In line with this, we plan to immediately monitor right-heart filling pressures through the PAC, together with PiCCO and MostCare monitoring systems, in our clinic. The absolute values of hemodynamic parameters such as CO, PAP, MAP, PCWP, and ITBV, regardless of the device used, are unsatisfactory and unsuitable for detecting the subtle instabilities of an LT recipient during a hyperdynamic state. Therefore, combining multiple sources of information may help physicians better interpret serious and otherwise obscure impairments in cardiovascular performance and volemic status and avoid conceptual and physiological pitfalls [27].

### Limitations

This study has some limitations. First, this was a retrospective study, with a possible risk of bias. However, the fact that both groups were similar with respect to demographic and clinical characteristics indicated that the risk of bias was minimized. Second, other specific hemodynamic patient parameters, aside from basic parameters such as arterial blood pressure, pulse, and saturation, were not recorded. That is, data regarding the average measurements of the variables in the monitoring systems could not be accessed. Prospective randomized studies and, if possible, multicenter studies are needed to validate our findings and address the limitations from the retrospective nature of our study.

## 5. Conclusions

The clinical and biochemical outcomes are not significantly different between PiCCO and MostCare PRAM as hemodynamic monitoring systems in LDLT. However, the MostCare system has important advantages such as being less invasive, requiring less equipment, being easy to use, and being cost-effective.

## Figures and Tables

**Table 1:** Comparison of clinicodemographic characteristics between the PiCCO and MostCare PRAM groups.

Parameters (Median (95% CI))	PiCCO Group (*n* = 90)	MostCare PRAM Group (*n* = 73)	*p*
Gender (%)			0.152
Female	25 (27.8)	28 (38.4)	
Male	65 (72.2)	45 (61.6)	
Recipient age (y)	53 (52–57)	56 (54–59)	0.188
BMI (kg/m^2^)	27 (25–29)	27 (26–29)	0.798
Donor age (y)	32 (30–35)	33 (30–35)	0.748
CHILD	8 (8–9)	8 (8–9)	0.074
MELD	14 (14–17)	16 (14–18)	0.175
EF	65 (65–67)	60 (60–65)	0.123
PAPS (mmHg)	30 (30–35)	30 (30–35)	0.068
Surgery time (h)	6.5 (6–7)	6 (6–7)	0.058
AHF (min)	60 (52–67)	55 (51–63)	0.460
CIT (min)	42 (38–45)	42 (39–46)	0.518
WIT (min)	37 (33–42)	41 (40–44)	0.695
ES (pice)	3 (3–4)	2 (2–3)	0.311
FFP (pice)	2 (2–3)	1 (1–2)	<0.001
PLT (pice)	1 (0–1)	1 (0–1)	0.037
HA (20% 100 mL)	8 (8–10)	7 (7–8)	0.135
Cristaloid (mL)	5500 (5000–6500)	5850 (5500–6000)	0.479
Urine output (mL)	2000 (2000–2500)	2000 (1600–2300)	0.598
GRWR	1.2 (1.2–1.4)	1.2 (1.2–1.4)	0.275
Hospital stay (d)	14 (13–17)	15 (12–16)	0.897
ICU stay (d)	1.5 (1–2)	1 (1–2)	0.060

BMI—body mass index; CHILD—Child–Pugh score; MELD—Model for End-Stage Liver Disease score; EF—ejection fraction; PAPS—systolic pulmonary artery pressure; AHF—anhepatic phase; CIT—cold ischemia time; WIT—warm ischemia time; ES—erythrocyte suspension; FFP—fresh frozen plasma; PLT—platelet; HA—human albumin; GRWR—graft to recipient weight ratio; ICU—intensive care unit.

**Table 2:** Changes in biochemical parameters from the day of surgery (postoperative (POD) 0) to POD 1, POD 3, and POD 7 after LT.

Parameters (Median (95% CI))	PiCCO Group (*n* = 90)	MostCare PRAM Group (*n* = 73)	*p*
PLT			
POD 0	64 (57–81)	74 (62–96)	0.113
POD 1	65 (57–70)	72 (65–86)	0.037
POD 3	54 (52–60)	58 (49–74)	0.730
POD 7	72 (64–86)	78 (65–91)	0.689
AST			
POD 0	156 (145–179)	162 (150–178)	0.680
POD 1	182 (164–209)	178 (153–205)	0.539
POD 3	78 (69–89)	61 (54–68)	0.008
POD 7	35 (32–44)	32 (27–37)	0.065
ALT			
POD 0	107 (100–129)	115 (104–128)	0.701
POD 1	174 (164–197)	188 (169–220)	0.502
POD 3	142 (126–157)	140 (122–159)	0.931
POD 7	74 (65–87)	70 (61–81)	0.823
Total Bilirubin			
POD 0	4.4 (4.0–5.5)	4.7 (4.0–5.5)	0.593
POD 1	5.6 (5.0–6.5)	5.3 (4.4–6.7)	0.783
POD 3	3.0 (2.2–3.3)	3.0 (2.3–3.6)	0.836
POD 7	2.0 (1.7–2.7)	1.7 (1.4–2.3)	0.271
Direct Bilirubin			
POD 0	1.2 (1.1–1.4)	1.4 (1.3–2.0)	0.200
POD 1	2.5 (2.1–2.9)	2.2 (1.5–3.0)	0.981
POD 3	1.6 (1.4–2.2.)	1.7 (1.3–2.2)	0.940
POD 7	1.4 (1.2–1.8)	1.2 (1.0–1.9)	0.454
PT			
POD 0	20 (19–21)	22 (20–23)	0.003
POD 1	20 (19–21)	21 (21–23)	0.009
POD 3	17 (16–18)	17 (17–18)	0.650
POD 7	14 (13–14)	13 (13–15)	0.416
INR			
POD 0	1.7 (1.6–1.8)	1.8 (1.8–2.0)	0.005
POD 1	1.7 (1.7–1.8)	1.8 (1.8–2.0)	0.001
POD 3	1.5 (1.4–1.5)	1.4 (1.4–1.5)	0.639
POD 7	1.1 (1.1–1.2)	1.1 (1.1–1.2)	0.175

PLT: platelet; AST: aspartate aminotransferase; ALT: alanine aminotransferase; PT: prothrombin time; INR: international normalized ratio.

## Data Availability

The datasets analyzed during the current study are available from the corresponding author on reasonable request.
